# Editorial: The use of video games and other technologies as intervention tools in neurodevelopmental disorders: perspectives and challenges

**DOI:** 10.3389/fnhum.2026.1785118

**Published:** 2026-02-11

**Authors:** Gabriella Medeiros Silva, Thiago P. Fernandes, Natanael Antonio dos Santos, Junya Fujino, Shisei Tei

**Affiliations:** 1 Laboratory of Perception, Neuroscience and Behavior, Federal University of Paraiba, João Pessoa, Brazil; 2 Department of Psychiatry and Behavioral Sciences, Graduate School of Medical and Dental Sciences, Tokyo Medical and Dental University, Tokyo, Japan; 3 Department of Psychiatry, Graduate School of Medicine, Kyoto University, Kyoto, Japan

**Keywords:** ADHD, autism, developmental disabilities, neurodevelopmental disorders, video games, virtual reality

Neurodevelopmental disorders (NDDs) are defined as a group of conditions characterized by early alterations in brain development, resulting in deficits in cognitive, behavioral, and social domains (Morris-Rosendahl and Crocq, 2020). NDDs may require interventions and adaptations to improve functionality and promote independence (Hadders-Algra, 2021). Despite advances in clinical and educational interventions, significant obstacles remain for promoting everyday functionality in individuals with NDDs.

In the clinical setting, long periods before visible progress and limited transfer of skills to everyday contexts can create difficulties in maintaining patient motivation (Koegel et al., 1998; Lamsal and Zwicker, 2017; Sandoval-Norton et al., 2019). In the educational context, more generic approaches that rely solely on neuropsychological aspects may be limited in promoting both inclusion and effective learning, as they lack integration with active teaching methodologies that place the student at the center of the learning process (Nadeau et al., 2020; Van der Merwe et al., 2020).

In this context, the use of technologies as intervention and teaching tools has grown. The literature indicates that playful resources, such as video games and other technologies, can enhance patients' attention and motivation (Clough and Casey, 2011; Silva et al., 2017). Accordingly, they may function as adjunct tools, stimulating brain areas important for cognitive and behavioral aspects (Silva et al., 2021).

In our Research Topic, “*The Use of Video Games and Other Technologies as Intervention Tools in Neurodevelopmental Disorders: Perspectives and Challenges*,” we present different technological resources (e.g., video games and virtual reality) as innovative and integrative tools in the context of educational and clinical interventions for neurodevelopmental disorders. Our aim was to gather new perspectives on practical applications and theoretical frameworks addressing how these technologies can be incorporated into educational and therapeutic programs to increase engagement, support socioemotional development, and promote the application of skills in real-life settings.

In the clinical context, three articles from our Research Topic analyzed aspects of the use of digital technologies in interventions. The scoping review by Glass and Galati examined the theoretical and practical aspects of designing and using games in the health domain. The authors highlighted a predominance of approaches grounded in psychological theories, with limited integration of game design principles, a critical point for intervention efficacy.

The narrative review by Doulou et al. identified that intervention protocols using virtual reality for children with NDDs exhibit high variability, including the number and duration of training sessions, as well as program characteristics (device, user perspective, level of immersion, and interactivity). The objectives of the studies also varied according to the target population. While most studies involving children diagnosed with attention-deficit/hyperactivity disorder (ADHD) focused on the assessment and training of attentional skills, studies with participants on the autism spectrum (ASD) prioritized social skills.

Stasolla et al. authored an opinion article proposing a three-step hierarchical solution for the assessment and intervention of individuals with NDDs. In the first step, a deep learning–based system would differentiate between individuals at risk for NDDs and typically developing individuals by mapping brain activity during cognitive tasks. The second step would involve proposing playful tasks, created based on the principles of Reinforcement Learning and gamification. The third step would address clinical validity, carried out by external evaluators who are experts in social validation procedures.

In the educational context, Videla, Aros, et al. developed a perspective article on the 3E approach (experience, interaction, and communication) for the educational inclusion of autistic students using technology. The work provides a critical reflection on neurodiversity from the 3E Cognition approach (embodied, enacted, and environmentally structured), which emphasizes brain–mind–environment interaction. From this perspective, digital technologies, such as virtual reality and video games, could be effective in the inclusion and teaching–learning processes for autistic children by enabling, for example, cues for social signals during interaction with the social environment or training in emotion recognition.

The same research group, Videla, Parada, et al., published another article on the topic, this time including cases illustrating how the 3E approach and technology can promote the inclusion of students with special educational needs. Case 1 described the use of a gamified application to support a 12-year-old student with ADHD in the context of environmental education. After using the application, significant improvements were observed in attention, self-regulation, and engagement. Case 2 reported the use of augmented reality and virtual reality to support language and mathematics teaching for 12 students aged 11–16. The immersive experiences provided greater engagement and motivation, as well as deeper understanding of the concepts.

From this perspective, the studies published in our Research Topic reinforce the potential of digital technologies as promising tools for both clinical rehabilitation and inclusive pedagogical practices in NDDs. However, they also highlight persistent challenges, such as the need for greater protocol standardization, ecological validation of outcomes, and effective integration of psychological, educational, and game design theories. Advancing in this direction will be crucial for these technologies to transcend their innovative character and establish themselves as evidence-based interventions centered on the real needs of individuals with NDDs.
